# Comparison of Clinical Balance and Visual Dependence Tests in Patients With Chronic Dizziness With and Without Persistent Postural-Perceptual Dizziness: A Cross-Sectional Study

**DOI:** 10.3389/fneur.2022.880714

**Published:** 2022-05-24

**Authors:** Charlotte De Vestel, Willem De Hertogh, Vincent Van Rompaey, Luc Vereeck

**Affiliations:** ^1^Department of Rehabilitation Sciences and Physiotherapy/Movant, Faculty of Medicine and Health Sciences, University of Antwerp, Antwerp, Belgium; ^2^Multidisciplinary Motor Centre Antwerp (M2OCEAN), Antwerp University Hospital, Antwerp, Belgium; ^3^Department of Otorhinolaryngology and Head & Neck Surgery, Antwerp University Hospital, Antwerp, Belgium; ^4^Department of Translational Neurosciences, Faculty of Medicine and Health Sciences, University of Antwerp, Antwerp, Belgium

**Keywords:** persistent postural-perceptual dizziness, chronic dizziness, vestibular diseases, balance, visual dependence

## Abstract

**Background:**

The diagnosis of persistent postural-perceptual dizziness (PPPD) is primarily based on medical history taking. Research on the value of clinical balance and visual dependence tests in identifying PPPD is scarce.

**Objectives:**

(1) to contrast clinical balance and visual dependence tests between PPPD patients, dizzy non-PPPD patients, and healthy persons; and (2) to evaluate whether these clinical tests can help to identify PPPD in patients with chronic dizziness.

**Methods:**

Consecutive patients with chronic dizziness (38 PPPD and 21 non-PPPD) and 69 healthy persons underwent Static Balance tests, the Timed Up and Go test, the Tandem Gait test, and the Functional Gait Assessment (FGA). Visual dependence tests included the Visual Vertigo Analog Scale (VVAS), the Rod-and-Disc test (RDT), and postural sway while facing rotating dots. Groups were compared using ANOVA with *post-hoc* Tukey, or independent samples *t*-tests. The value of the clinical tests for PPPD identification was evaluated through logistic regression and Partial Least Squares Discriminant (PLS-DA) analyses.

**Results:**

PPPD patients had significantly higher VVAS scores than dizzy non-PPPD patients (*p* = 0.006). Facing rotating dots, PPPD and dizzy non-PPPD patients had increased postural sway compared to healthy persons (PPPD vs. healthy: center of pressure (COP) velocity *p* < 0.001, and COP area *p* < 0.001; but non-PPPD vs. healthy: COP velocity *p* = 0.116 and COP area *p* = 0.207). PPPD patients had no significantly increased postural sway compared to dizzy non-PPPD patients. PPPD and dizzy non-PPPD patients also scored significantly worse on balance tests compared to healthy persons (PPPD vs. healthy: for all balance tests *p* < 0.001; non-PPPD vs. healthy: FGA *p* < 0.001, for all other tests *p* < 0.05). Differences were insignificant in balance scores between PPPD and dizzy non-PPPD patients, or in RDT scores between the three study groups. In patients with chronic dizziness, a higher VVAS score was most associated with PPPD [odds ratio 1.04; 95% CI (1.01; 1.07); *p* = 0.010]. The cross-validated (CV) PLS-DA model with all clinical tests included, had fair discriminative ability (CVerror = 47%).

**Conclusion:**

PPPD patients were more visually dependent, but did not have worse postural balance compared to dizzy non-PPPD patients. Elevated VVAS scores characterized PPPD most in patients with chronic dizziness.

## Introduction

With a prevalence of up to 20%, persistent postural-perceptual dizziness (PPPD) is among the top five most common causes of vestibular complaints reported in tertiary care hospitals (1–3). PPPD is designated by the Bárány Society as a separate vestibular disorder and an umbrella term for four subtypes: Phobic Postural Vertigo, Space-Motion Discomfort, Visual Vertigo, and Chronic Subjective Dizziness (4).

Patients with PPPD clinically present with non-rotatory vertigo and postural imbalance, which are present almost on a daily basis. The symptoms are worsened by upright posture, active or passive movements, and visual stimuli (4). Visual stimulation is the most characteristic aggravating factor for PPPD (5).

The pathophysiological mechanism of PPPD and its four subtypes is still uncertain (6). It is thought to result from maladaptation to a condition that caused vestibular symptoms (e.g., a peripheral or central vestibular disorder, vestibular migraine, or psychogenic dizziness) (6). Previous research identified altered functional brain connectivity (7, 8): i.e. reduced between the (pre)cuneus and the premotor cortex (8), and increased in the visual cortices (9). The former impairs the regulation of body posture and movement (10, 11), while the latter leads to increased visual dependence (12). Excessive reliance on visual information often causes dizziness and/or postural instability in visually disturbing situations (13).

The diagnosis of PPPD is currently primarily based on medical history taking (4). The often vague symptoms in patients with chronic dizziness tend to correlate weakly with the results of standard vestibular tests, making diagnosis difficult (14).

Several clinical tests exist in the literature that allow for evaluation of postural balance and visual dependence in patients with a vestibular disorder (13, 15). However, it is not yet clear whether these clinical tests can be used for identifying PPPD in patients with chronic dizziness.

The aim of this study was 2 fold: (1) to contrast clinical balance and visual dependence tests between PPPD patients, dizzy non-PPPD patients, and healthy persons; and (2) to evaluate whether these clinical tests can help to identify PPPD in patients with chronic dizziness.

## Materials and Methods

### Design and Setting

This study is a cross-sectional study consisting of consecutive patients enrolled between March 2019 and July 2020, either at the Department of Otolaryngology of the Antwerp University Hospital or in one of the two participating general hospitals (AZ Klina, Brasschaat and AZ Sint-Jozef, Malle). The control group consisted of healthy persons from the direct (employees) or indirect environment (family and friends) of the MOVANT research team. The study was performed in the M2OCEAN laboratory (Multidisciplinary Motor Centre Antwerp) of the Antwerp University Hospital.

The study report is drafted conform the “Strengthening the Reporting of Observational studies in Epidemiology (STROBE)” guidelines for cross-sectional studies (16).

### Ethical Considerations

Ethical approval was obtained from the Medical Ethics Committees of the Antwerp University Hospital (reference number 18/586).

### Participants

Patients' eligibility was assessed by an Ear-Nose-Throat (ENT) specialist through medical history taking [using the SO STONED method (17)], and through a micro-otoscopic, a vestibular (including video head impulse, sinusoidal harmonic acceleration, and binaural bithermal caloric testing) and an audiometric assessment. The inclusion criteria were: (1) speaking the Dutch language; (2) being at least 18 years old; and (3) suffering of chronic non-rotatory vertigo and/or unsteadiness for at least 15 days per month for a minimum of 3 months. The exclusion criteria were: (1) presence of an acute vestibular disorder; (2) balance problems not due to dizziness (e.g., neurological, orthopedic, or other medical conditions); (3) dizziness due to untreated metabolic or cardiac disease, hormonal disturbances, vasovagal syncope, hyperventilation, acute mental problems, or substance abuse; and (4) severe visual impairment, not correctable by e.g., wearing glasses.

Eligibility of healthy persons was verified by the researcher. Their inclusion criteria were (1) speaking the Dutch language; and (2) being at least 18 years old. The exclusion criteria were: (1) history of or currently suffering from rotatory vertigo; (2) frequent episodes of non-rotatory vertigo (more than one episode in 3 months); (3) balance problems; and (4) severe visual impairment, not correctable by e.g., wearing glasses.

### Diagnosis of PPPD

A patient was diagnosed with PPPD if he or she met all five diagnostic criteria for PPPD as established by the Committee for Classification of Vestibular Disorders of the Bárány Society (4). These are: (1) presence of chronic (≥ 3 months) non-spinning (rotatory) vertigo, dizziness or unsteadiness; (2) symptoms are aggravated by an upright position, active/passive body movements, and visual stimuli; (3) prior presence of a condition that caused dizziness or instability; (4) symptoms have a major impact on patients' mental or physical functioning; and (5) symptoms cannot be explained by another existing condition.

### Outcome Variables

#### Descriptive Variables

*Age (years), gender, dizziness duration (years)*, and *ENT diagnosis* were collected from the electronic patient record.

The *Dizziness Handicap Inventory (DHI)* evaluates the physical, functional and emotional handicap experienced by patients as a result of their vestibular symptoms. For 25 statements, patients were asked to indicate the extent to which they applied to them (“no” = 0; “sometimes” = 2; “yes” = 4 points). The total DHI score was recorded, ranging from 0 (no impairment) to 100 (maximal impairment) (18, 19).

The *Hospital Anxiety and Depression Scale (HADS)* evaluates patients' emotional state by means of 7 anxiety-related (HADS-A) and 7 depression-related (HADS-D) questions answered on a 4-level ordinal scale. The total HADS-A and HADS-D scores, both ranging from 0 (no anxiety/depression) to 21 (maximal anxiety/depression), were retained (20).

**Figure 1:**
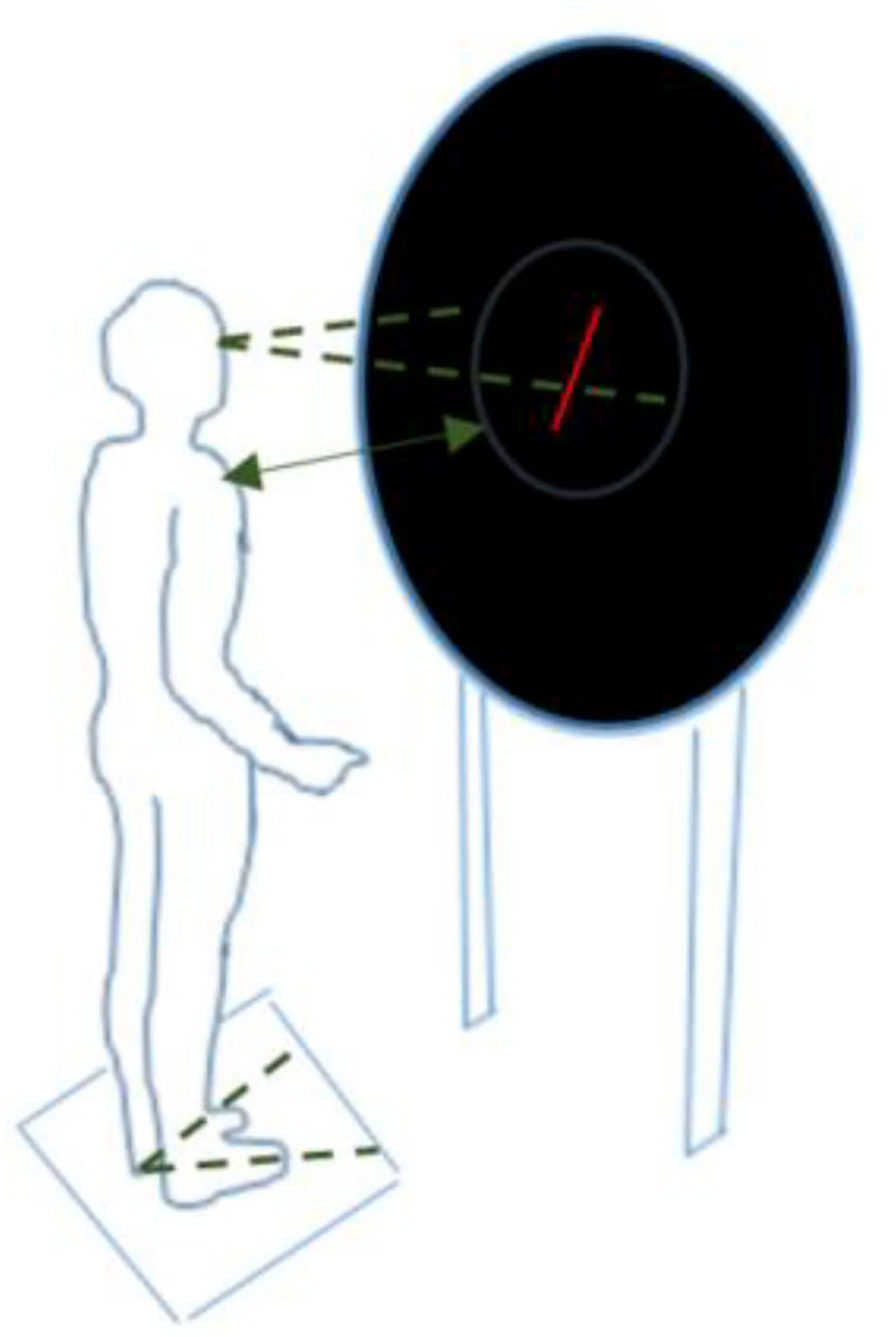
Experimental setup for the Subjective Visual Vertical test, Rod-and-Disc test, and postural sway while facing rotating dots (screen display varied depending on the test condition) ^a, b^. ^a^The participant stood upright, barefoot, with arms alongside the body, in a completely dark room. A television screen was placed at eye level at a distance of 40 cm, providing an almost full-field stimulus of 80%. A ring with an inner diameter of 54.5 was mounted on the television set to prevent the edges of the television screen from acting as a frame of reference. ^b^The feet were placed at an angle of 20 degrees with the inner malleoli 10 cm apart.

The *Subjective Visual Vertical (SVV) test* measures patients' perception of verticality in the absence of visual reference points. The experimental setup is shown in Figure 1. The patient was asked to reposition a red line (6 cm length) on a black background until they felt it matched the true vertical. This was done using a handheld remote control and without moving the head or body. The test was done twice with the line initially tilted 20 degrees to the left, and twice with the line tilted 20 degrees to the right. More information on the test conditions can be found in the (Supplementary Table 1). Performance was expressed as the mean absolute misalignment (in degrees) with the gravitational vertical (0°) for these four tests.

#### Clinical Balance Variables

The *Static Balance tests* measures the patients' balance while they were standing still and upright in four different foot positions with eyes closed: (1) feet together, combined with Jendrassik maneuver (i.e., fingers were interlocked with arms in abduction, and tension was created by pulling the hands apart); (2) feet 5 cm apart standing on a foam plate (NeuroCom International Inc., Clackamas, USA; 60 kg/cm^3^ medium density; 45 × 45 × 12 cm), combined with Jendrassik maneuver (i.e., the same hand grip as described above); (3) heel-to-toe tandem stance; and (4) standing on one leg. For each condition, the patient had three attempts to maintain the respective condition 30 s. Only the best score (in seconds) out of these three attempts was retained. The sum of these best scores for each of the four conditions constitutes the total static balance score, which ranges from 0 (markedly reduced balance) to 120 (excellent balance) (15).

The *Timed Up and Go test* evaluates how quickly a person can get up from a chair, walk 3 m, turn around, walk back and sit back down on the chair. The fastest performance time (in seconds) of three attempts was retained (15).

The *Tandem Gait test* measures the number of correctly performed steps when walking heel-to-toe on a straight line. The highest number of steps of three attempts was retained. The maximal score was 20 steps (15).

The *Functional Gait Assessment (FGA)* evaluates balance control during ten different walking tasks (e.g., walking fast, or walking with head movements). The total FGA score ranges between 0 (markedly reduced balance) and 30 (normal balance) (21).

#### Clinical Visual Dependence Variables

The *Visual Vertigo Analog Scale (VVAS)* is a questionnaire consisting of nine statements describing different situations in daily life where disturbing visual stimuli are present (e.g., supermarket, or traffic at a busy intersection). For each statement, patients were asked to mark the extent to which they experienced dizziness on a 10 cm visual analog scale. If a situation was not applicable for the patient, it was not marked and not included in the final score. The total VVAS score is the sum of the marks for each relevant situation, rescaled to a percentage where 100% indicates maximal visually induced dizziness (22).

The *Rod-and-Disc test (RDT)* measures the influence of visual disturbance on the perception of verticality. The experimental setup and the four test conditions were the same as for the SVV test, but for the RDT each test had to be performed twice with dots rotating clockwise (CW) and twice with dots rotating counter-clockwise (CCW) on the black screen. More information on the test conditions can be found as Supplementary Table 1. The mean misalignment for the SVV test was used as baseline and subtracted from the differences in misalignment with the gravitational vertical (0°) for each of the CW and CCW rotating dots tests. These adjusted differences were then averaged out (after inverting the sign of the data for the CCW rotating dots, as the directions were mirrored), resulting in an indication of the overall impact of visual disturbance on the perception of verticality. A positive value means that the patient placed the line more in the direction of the rotating dots compared to the SVV, a negative value more opposite to the direction of the rotating dots.

Preliminary analyses showed that the Rod-and-Frame test-which was equivalent to the RDT, except that the dots were replaced by a frame tilted left or right-yielded less pronounced results than the RDT, and is therefore not retained for discussion. The test conditions and data are available as Supplementary Tables 2, 3.

*Postural sway while facing rotating dots* was evaluated by measuring the displacement of patients' center of pressure (COP) first while looking at a black screen, and then during exposure to dots rotating CW and dots rotating CCW. The experimental setup differed from the SVV and RDT that no line was shown in any of the three tests, and that the patient stood on a force plate (AMTI type OR 6; 1,000 fps, 46 × 50 × 8 cm). More information on the test conditions can be found as Supplementary Table 1. Postural sway parameters for COP lean, COP velocity, and COP area were computed. The degree of visual dependence was calculated by subtracting the baseline value (black screen condition) from the values obtained during the CW and CCW rotating dots tests. More information is provided in Table 1.

**Table 1:** Parameters of the postural sway while facing rotating dots^a^.

**Postural sway parameters**	**Description**
COP lean (mediolateral; mm)	Average deviation of the COP in mediolateral direction in relation to the starting position. Data were reversed for CCW rotating dots to average the data for CW and CCW rotating dots, resulting in an indication of the overall impact of visual disturbance on the COP lean. A positive value means that the body leaned in the direction of the rotating dots, a negative value indicates leaning in the opposite direction.
COP velocity (mediolateral; mm/s)	Average velocity of the COP in mediolateral direction. A positive value means that the COP velocity was larger during the rotating dots conditions compared to the baseline condition (black screen), while a negative value means the opposite.
COP area (mm^2^)	Ellipse that contains 85% of the COP data. A positive value means that the COP area was larger during the rotating dots conditions compared to the baseline condition (black screen), while a negative value means the opposite.

^a^*COP, center of pressure; mm, millimeters; s, seconds*.

Preliminary analyses showed that the postural sway while facing a tilted frame–which was equivalent to the postural sway while facing rotating dots, except that the dots were replaced by a frame tilted left or right-yielded less pronounced postural sway results compared to the rotating dots conditions, and are therefore not retained for discussion. The test conditions and data are available as Supplementary Tables 2, 3.

### Data Sources and Measurement

The researcher is an accredited physiotherapist (Master's degree). The study took 2.5 h per participant, using the following strict assessment order: first the questionnaires, then the clinical balance tests, and finally the visual dependence tests. The visual dependence tests (except the VVAS) were performed in a different randomized order for every participant to avoid a habituation effect on the results. Breaks were allowed between and during the different tests. The next test was only started when the dizziness symptoms had returned to participant's baseline level. The data were collected pseudonymised in an IBM SPSS Statistics data file stored on a secure server of the University of Antwerp.

### Blinding

During data collection, the researcher was blinded to the ENT diagnosis of each patient. Blinding to whether a participant was a patient or healthy person was not possible, as the healthy persons were recruited from the environment of the research team.

### Statistical Analyses

Descriptive statistics included means and standard deviations for quantitative variables, and frequencies and percentages for categorical variables. Shapiro-Wilk tests indicated whether quantitative variables were normally distributed (23).

Inter-group comparison of quantitative variables was carried out through one-way analysis of variance (ANOVA), using a Bonferroni-corrected significance level of *p* < 0.006 accounting for 9 comparisons, followed by *post-hoc* analysis with Tukey correction for multiple testing. For inter-group comparison of the categorical variables, chi-square (χ2) tests were used. Two-group comparison of dizziness-related variables was carried out through independent samples *t*-tests.

Univariable logistic regression and Partial Least Squares Discriminant Analysis (PLS-DA) models were fitted to determine the predictors of PPPD in patients with chronic dizziness. The relation between the clinical tests and PPPD was expressed as odds ratios [95% confidence interval (CI)], area under the receiver operating characteristic curve (AUC), and optimal cut-off with corresponding sensitivity and specificity (according to maximization of the Youden index). For the PLS-DA model, the potential of each of the clinical tests to discriminate between PPPD and dizzy non-PPPD patients was evaluated through their loading values. The overall discriminative value of the PLS-DA model was 5-fold cross-validated (CV) (24). In brief, for each fold we included 80% of the observations in the training set and 20% in the validation set. The training set was used to fit the PLS-DA models, with the number of components ranging from 2 to 10. Subsequently, these models predicted the outcome from the observations in the validation set, and the percentage of error (CVerror) was registered. This was carried out in 5-fold, with each individual observation belonging to the validation set exactly one time. The 5-fold CV was repeated 500 times to obtain the standard error of the CVerror.

Significance was set at *p* < 0.05, unless otherwise stated. Statistical analyses were performed using the SPSS software version 27.0 (25). The PLS-DA was fitted with the R software (26).

## Results

### Participants

Seventy patients with chronic dizziness met the predefined study eligibility criteria as listed in the method section. Nine patients decided not to participate because of reduction of dizziness complaints with medication and/or physical therapy (2 patients), personal reasons (3 patients), or COVID outbreak (4 patients). The results of two other patients had to be excluded after the study because of unreliable test results due to disturbing background noise near the laboratory during the testing.

Of the 59 patients (mean age 57.34 ± 12.96) that successfully participated, 38 were diagnosed with PPPD. The 21 patients without PPPD were primarily diagnosed with vestibular hypofunction (4 patients), bilateral vestibulopathy (1 patient), proprioceptive cervicogenic dizziness (8 patients), multiple sensory deficits (3 patients), and mal de débarquement syndrome (1 patient). In four patients no cause could be found.

The control group consisted of 69 healthy non-dizzy persons (mean age 51.71 ± 17.24).

**Figure 2:**
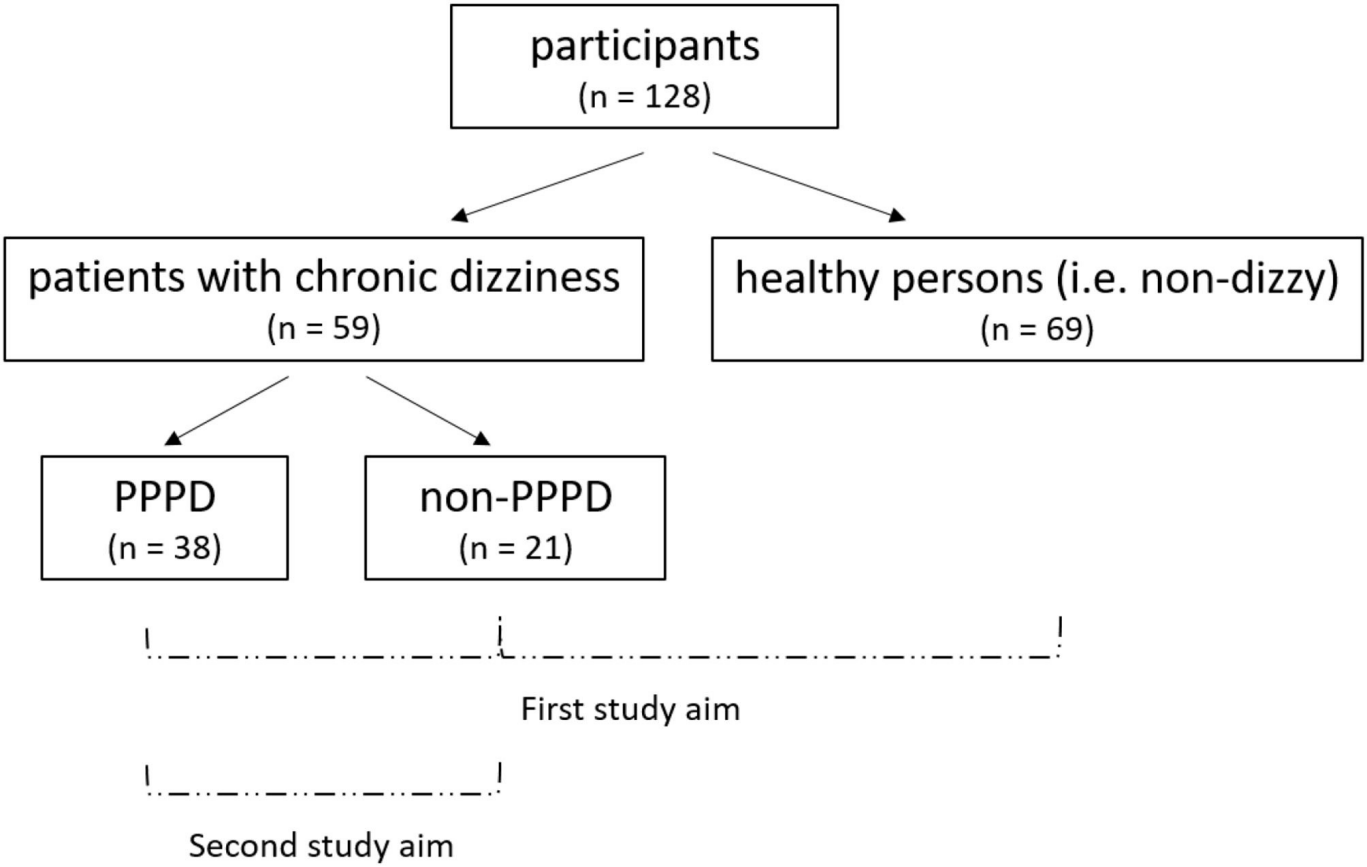
Overview of the recruited samples and study aims^a^. First study aim: comparison of the clinical balance and visual dependence tests between PPPD patients, dizzy non-PPPD patients, and healthy persons. Second study aim: evaluation whether these clinical tests can help to identify PPPD in patients with chronic dizziness. ^a^PPPD, persistent postural-perceptual dizziness.

An overview of the recruited samples and the study aims are shown in Figure 2.

### Sample Characteristics

Gender numbers (*p* = 0.453) were not significantly different between the three study groups.

All patients with chronic dizziness had significantly higher HADS-A (PPPD vs. healthy, *p* < 0.001; dizzy non-PPPD vs. healthy, *p* = 0.020) and HADS-D scores (PPPD vs. healthy, *p* < 0.001; dizzy non-PPPD vs. healthy, *p* < 0.001) compared to healthy persons. PPPD patients had significantly higher SVV scores (*p* = 0.016) and dizzy non-PPPD patients were significantly older (*p* = 0.037), both compared to healthy persons.

PPPD patients had significantly higher duration of dizziness complaints (*p* = 0.003), but not significantly higher DHI (*p* = 0.065), HADS (HADS-A, *p* = 0.308; HADS-D, *p* = 1.00) and SVV scores (*p* = 0.406), compared to dizzy non-PPPD patients.

Data are provided in Table 2.

**Table 2:** Results on descriptive variables of the included participants ^a, b^.

	**Mean** **±SD or number (%)**			**Statistical analyses (** ***p*** **-values)**			
**Measurement tool (unit)**	**Chronic dizziness**		**Healthy** **(*n* = 69)**	**3-group comparison**	**With PPPD vs. without PPPD**	**With PPPD vs. healthy**	**Without PPPD vs. healthy**
	**With PPPD** **(*n* = 38)**	**Without PPPD** **(*n* = 21)**					
Age (years)	55.18 ± 12.31	61.23 ± 13.49	51.71 ± 17.24	**0.045** ^**†**^ ^*^	0.320	0.503	**0.037** ^¶^^*^
Female	24 (63.2)	10 (47.6)	34 (49.3)	0.453^§^			
Dizziness duration (years)	7.84 ± 6.50	3.91 ± 3.24	N/A		**0.003** ^**‡**^ ^*^		
DHI (0–100)	46.32 ± 18.18	36.95 ± 18.59	N/A		0.065^‡^		
HADS_anxiety_ (0–21)	7.21 ± 3.89	5.86 ± 4.00	3.32 ± 2.25	**<0.001** ^**†**^ ^**^	0.308^¶^	**<0.001** ^¶^^**^	**0.020** ^¶^^*^
HADS _depression_ (0–21)	5.68 ± 3.43	5.67 ± 3.62	1.57 ± 1.63	**<0.001** ^**†**^ ^**^	1.000^¶^	**<0.001** ^¶^^**^	**<0.001** ^¶^^**^
SVV (^°^)	1.73 ± 1.17	1.38 ± 0.90	1.18 ± 0.84	**0.022** ^**†**^ ^*^	0.406 ^¶^	**0.016** ^¶^^*^	0.707^¶^

^a^ *ANOVA test*

(†) ,*Independent samples t-test*

(‡) ,*Chi-squared test*

(§) ,*post-hoc analysis with Tukey correction (¶)*.

ANOVA: p < 0.05 (^*^) and p < 0.001 (^**^).

° ^b^, *degrees; DHI, Dizziness Handicap Inventory; HADS, Hospital Anxiety and Depression Scale; PPPD, persistent postural-perceptual dizziness; SD, standard deviation; SVV, Subjective Visual Vertical; vs, versus*.

### Comparison of the Clinical Balance Tests Between PPPD Patients, Dizzy Non-PPPD Patients, and Healthy Persons

All patients with chronic dizziness had significantly worse static (PPPD vs. healthy, *p* < 0.001; dizzy non-PPPD vs. healthy, *p* = 0.002) and dynamic balance scores (Timed Up and Go, Tandem Gait, and FGA, PPPD vs. healthy, *p* < 0.001; dizzy non-PPPD vs. healthy, *p* < 0.05) compared to healthy persons.

Between PPPD and dizzy non-PPPD patients, no significant differences were found in either static (*p* = 0.680) or dynamic balance scores (Timed Up and Go, *p* = 0.846; Tandem Gait, *p* = 0.954; and FGA, *p* = 0.813).

Data are provided in Table 3.

**Table 3:** Results on clinical balance tests of the included participants ^a, b^.

	**Mean** **±SD**			**Statistical analyses (** ***p*** **-values)**			
**Measurement tool (unit)**	**Chronic dizziness**		**Healthy** **(*n* = 69)**	**3-group comparison**	**With PPPD vs. without PPPD**	**With PPPD vs. healthy**	**Without PPPD vs. healthy**
	**With PPPD** **(*n* = 38)**	**Without PPPD** **(*n* = 21)**					
Static balance test (0–120 s)	64.22 ± 33.07	70.32 ± 27.09	93.30 ± 22.49	**<0.001** ^†**^	0.680^¶^	**<0.001** ^¶***^	**0.002** ^¶*^
Timed Up and Go test (s)	8.07 ± 2.70	7.78 ± 2.57	6.34 ± 1.13	**<0.001** ^†**^	0.846^¶^	**<0.001** ^¶***^	**0.012** ^¶*^
Tandem gait (# steps)	13.47 ± 7.95	13.95 ± 8.23	18.84 ± 3.60	**<0.001** ^†**^	0.954^¶^	**<0.001** ^¶***^	**0.004** ^¶*^
FGA (0–30)	23.24 ± 4.38	23.81 ± 5.17	28.25± 1.82	**<0.001** ^†**^	0.813^¶^	**<0.001** ^¶***^	**<0.001** ^¶***^

^a^*ANOVA test (†), post-hoc analysis with Tukey correction (¶)*.

ANOVA Bonferroni cut-off: p < 0.006 (**); other tests: p < 0.05 (*) and p < 0.001 (***).

^b^*#, amount; FGA, Functional Gait Assessment; PPPD, persistent postural-perceptual dizziness; SD, standard deviation; TUG, Timed Up and Go test; vs, versus*.

### Comparison of the Clinical Visual Dependence Tests Between PPPD Patients, Dizzy Non-PPPD Patients, and Healthy Persons

There was no significant difference in RDT results (*p* = 0.431) between the three study groups. The RDT values were positive in all three groups, indicating that in all cases the perceived vertical had an larger offset in the same direction of the rotation of the dots compared to the SVV tests. The COP lean results (*p* = 0.800) were not significantly different between the three study groups either. The COP lean values were small ( ≤ 1 mm), indicating that the visual disturbance did not cause the participant to tilt more to one side.

Patients suffering from chronic dizziness had larger COP velocity values (PPPD vs. healthy, *p* < 0.001; dizzy non-PPPD vs. healthy, *p* = 0.116) compared to healthy persons. The COP velocity values were positive in all three study groups, indicating larger mediolateral sway in visually disturbing conditions compared to the SVV tests. Next to this, PPPD patients also showed a significantly higher COP area (*p* < 0.001) compared to healthy persons. The COP area values were positive in all three study groups, indicating that the COP displacement was larger in visually disturbing conditions compared to the SVV condition.

PPPD patients had significantly higher VVAS scores (*p* = 0.006), but not significantly larger COP velocity values (*p* = 0.475) and COP area values (*p* = 0.116), compared to dizzy non-PPPD patients.

Data are provided in Table 4.

**Table 4:** Results on clinical visual dependence tests of the included participants ^a, b^.

	**Mean** **±SD**			**Statistical analyses (** ***p*** **-values)**			
**Measurement tool (unit)**	**Chronic dizziness**		**Healthy** **(*n* = 69)**	**3-group comparison**	**With PPPD vs. without PPPD**	**With PPPD vs. healthy**	**Without PPPD vs. healthy**
	**With PPPD** **(*n* = 38)**	**Without PPPD** **(*n* = 21)**					
VVAS (0–100%)	33.87 ± 20.65	18.13 ± 20.08	N/A		**0.006** ^‡*^		
RDT (degrees)	6.05 ± 3.96	6.09 ± 3.80	5.07 ± 4.21	0.431^†^			
Postural sway analysis with rotating dots							
COP lean (mm)	−0.05 ± 10.92	−0.32 ± 6.28	−1.01 ± 4.95	0.800^†^	0.475^¶^	**<0.001** ^¶***^	0.116^¶^
COP velocity (mm/s)	11.04 ± 15.32	7.68 ± 11.75	2.36 ± 5.12	** <0.001** ^†**^	0.116^¶^	** <0.001** ^¶***^	0.207^¶^
COP area (mm^2^)	648.44 ± 1009.44	302.91 ± 648.15	31.38 ± 132.36	**<0.001** ^†**^			

^a^*ANOVA test (†), Independent samples t-test (‡), post-hoc analysis with Tukey correction (¶)*.

ANOVA Bonferroni cut-off: p < 0.006 (**); other tests: p < 0.05 (*) and p < 0.001 (***).

^b^*COP, center of pressure; mm, millimeters; PPPD, persistent postural-perceptual dizziness; RDT, Rod-and-Disc test; s, seconds; SD, standard deviation; vs, versus; VVAS, Visual Vertigo Analog Scale*.

### Evaluation of the Value of the Clinical Tests for PPPD Identification in Patients With Chronic Dizziness

Univariable logistic regression analyses of the clinical tests, displayed in Table 5, revealed that a higher VVAS score was associated most with PPPD [odds ratio 1.04; 95% CI (1.01; 1.07); *p* = 0.010].

**Table 5:** Univariable logistic regression of the clinical tests for the prediction of PPPD in patients with chronic dizziness ^a, b^.

**Measurement tools**	**Estimate**	**Standard error**	**OR (95% CI)**	***p*-value**
Static Balance tests (0–120 s)	−0.007	0.009	0.99 [0.98; 1.01]	0.467
Timed Up and Go test (s)	0.045	0.109	1.05 [0.85; 1.29]	0.678
Tandem Gait (# steps)	−0.008	0.035	0.99 [0.93; 1.06]	0.824
FGA (0–30)	−0.027	0.060	0.97 [0.87; 1.10]	0.648
VVAS (0–100%)	0.039	0.015	1.04 [1.01; 1.07]	0.010*
RDT (°)	−0.003	0.076	1.00 [0.87; 1.16]	0.968
Postural sway while facing rotating dots				
COP lean (mm)	0.003	0.030	1.00 [0.95; 1.06]	0.918
COP velocity (mm/s)	0.019	0.023	1.02 [0.97; 1.07]	0.404
COP area (mm^2^)	0.001	<0.001	1.00 [1.00; 1.001]	0.197

^a^p < 0.05 (*).

^b^*COP, center of pressure; FGA, Functional Gait Assessment; mm, millimeters; OD, odds ratio; PPPD, persistent postural-perceptual dizziness; RDT, Rod-and-Disc test; s, seconds; TUG, Timed Up and Go test; vs, versus; VVAS, Visual Vertigo Analog Scale*.

The ROC analysis of the VVAS showed fair discriminative accuracy [AUC = 0.72, 95% CI (0.57; 0.86)]. The cut-off value for the VVAS (through maximization of the Youden index) was 21.01 which resulted in a sensitivity of 0.74 and a specificity of 0.67 for identification of PPPD.

**Figure 3:**
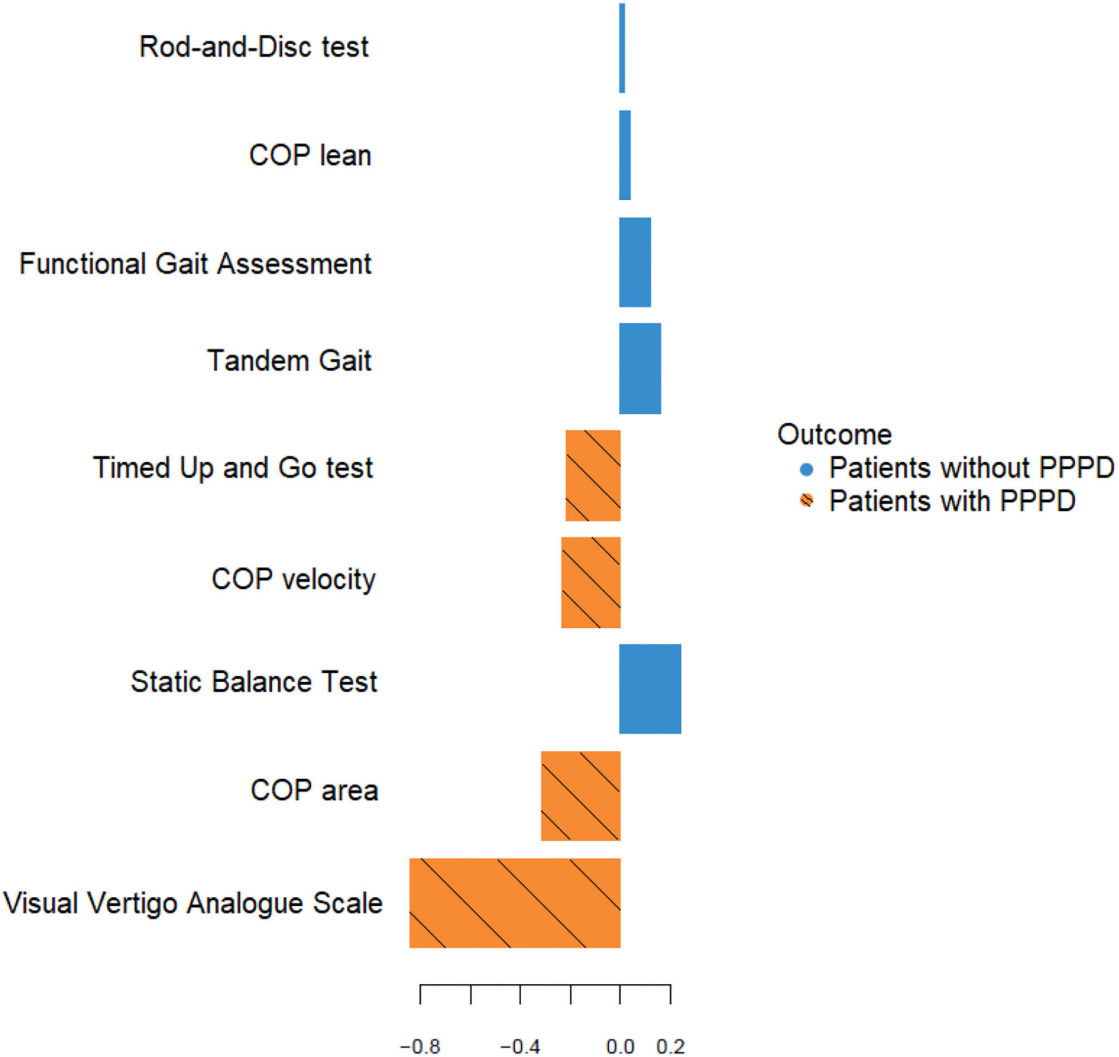
Loading plot of the first component of the PLS-DA model ^a, b^. ^a^Loadings of the original variables for the first PLS-DA component are represented on the X-axis. The color of the horizontal bars shows in which of the two groups (patients with or without PPPD) the mean value of the original variable is the largest. In this dataset, the original variables with a negative loading on the first PLSDA component all have a larger mean value in the patients with PPPD. ^b^COP, center of pressure; PPPD, persistent postural-perceptual dizziness.

In addition, a PLS-DA model was built to classify patients with chronic dizziness into PPPD and non-PPPD based upon all clinical tests listed in Table 5. The highest loading in the first component of the PLS-DA model was observed for VVAS, which is in line with the result from the logistic regression analysis where VVAS showed the strongest positive association with PPPD. Loadings are graphically shown in Figure 3.

**Figure 4:**
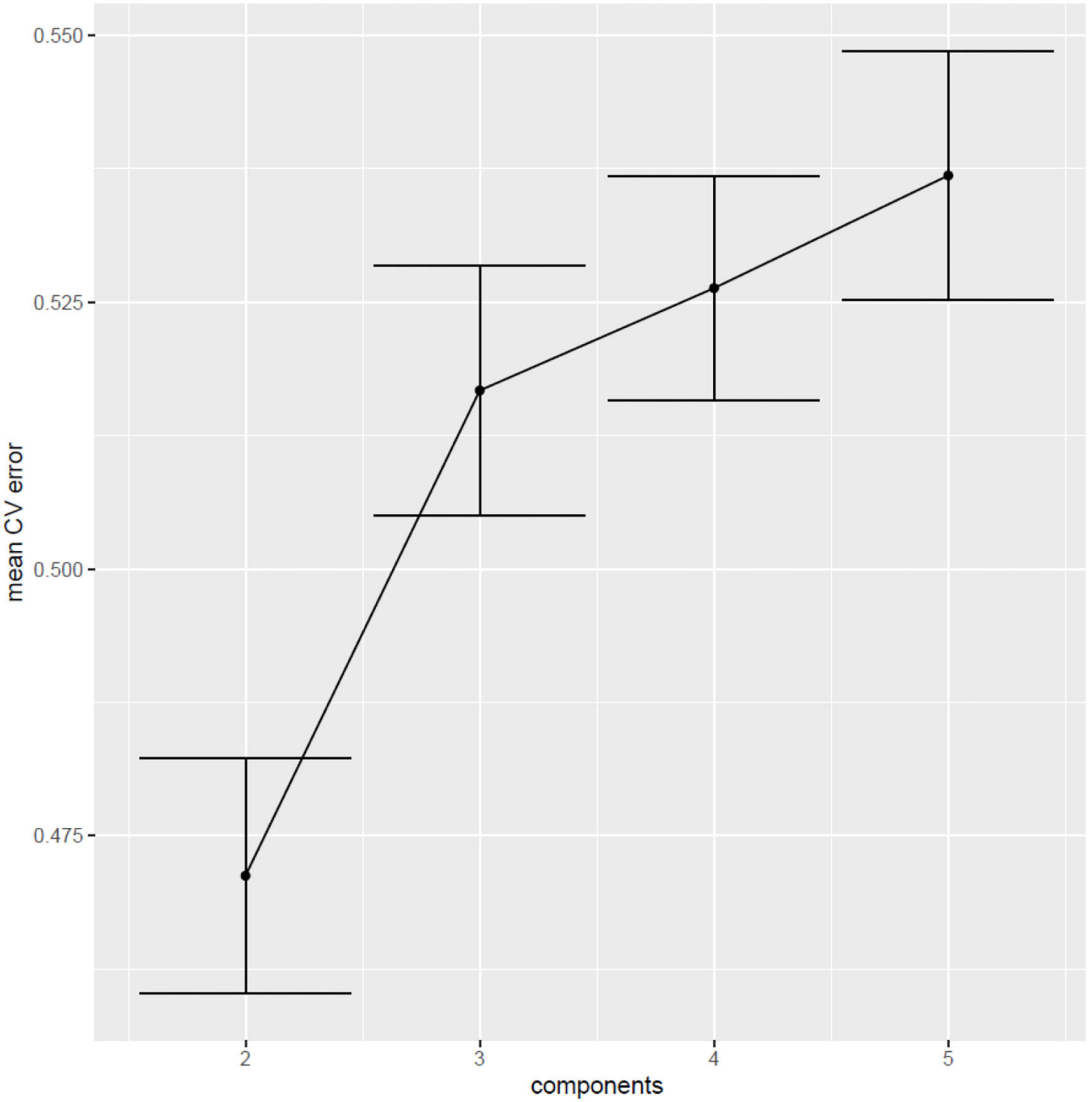
Cross-validation error (5-fold) of 500 iterations ^a, b^. ^a^This figure shows the cross-validation error of the models vs. the number of components. The error bars represent the 95% confidence interval around the mean CVerror (across 500 runs of 5-fold CV). The best performance is observed in the models with 2 components, with a CVerror of 47% [95% CI (0.46; 0.48)].^b^ CV, cross-validation.

The first two components of the PLS-DA accounted for 51% of the variance in the data (Kappa coefficient = 0.32; AUC of the ROC curve = 0.70). However, since the same observations were used to build the model and to test its predictive power, the prediction error is probably underestimated. To obtain an unbiased estimate of the prediction error of the PLS-DA model, and to find the optimal number of components to be included, 5-fold cross validation (CV) was carried out. The CVerror was 47%, as presented in Figure 4, which indicates that the PLS-DA model performs fairly in distinguishing between PPPD patients and dizzy non-PPPD patients.

## Discussion

Our results show significantly reduced static and dynamic balance scores in patients with chronic dizziness compared to healthy subjects. However, no significant difference in any balance test results could be demonstrated between patients with and without PPPD. The literature indeed confirms reduced balance in all forms of chronic dizziness (6, 27–29). This is attributed to ongoing sensory conflict between the visual, vestibular and proprioceptive systems that are responsible for maintaining postural balance (30). Additionally, emotions (e.g., fear can cause conditioned avoidance) (31, 32) and musculoskeletal pain or dysfunction (33) are known triggers for balance problems. None of these triggers are specific to PPPD.

Furthermore, our results show increased postural sway while facing rotating dots compared to the baseline condition (black screen) in all three study groups, as indicated by the positive values for COP velocity and COP area. The largest increase in postural sway was found in PPPD patients. These results are in line with the literature which states that disturbing visual stimuli can reduce balance control, especially in persons who are less able to rely on their vestibular and proprioceptive systems in visually challenging conditions (i.e., increased visual dependence) (34). In PPPD patients, this increased visual dependence can be expected as it is one of the diagnostic criteria of PPPD (4). Increased visual dependence also occurs in patients with chronic dizziness of non-PPPD origin as it is a frequently used coping mechanism for reduced vestibular function, aimed at restoring postural balance (35–37). On top of this, there is an inter-individual variability in visual susceptibility in the general population, which explains why participants without dizziness or balance complaints can have increased postural sway in visually challenging conditions (38).

The COP lean deviations were very limited ( ≤ 1 mm), but in line with the low values reported in the literature (13), and not significantly different between the three groups. The lean effect could be strengthened in future research by: (1) only showing rotating dots in the peripheral field while keeping the center of the screen empty (13, 34), or (2) by showing more realistic visual materials (e.g., fairground carousel, passing traffic) (39).

Another finding was the larger SVV deviations in all patients with chronic dizziness (both PPPD and non-PPPD patients) compared to healthy persons, although in none of the three study groups the SVV values were pathological [i.e., they were within the normal limit of 2.5° (40–43)]. The literature indicates that the upright SVV is sufficiently compensated in the chronic vestibular phase (44–46).

In our results, the SVV misalignment of RDT was not significantly different in PPPD patients compared to non-PPPD patients. This result agrees with earlier findings indicating that SVV misalignment of RDT is independent of the subtype of chronic dizziness, e.g., no significant differences in scores were found between visual vertigo and labyrinth-defective subjects (13), or between patients with vestibular neuritis with high vs. low DHI scores (35). In contrast to other studies, we could not observe significant differences in SVV misalignment of RDT between dizzy patients and healthy subjects (13, 35). The higher SVV errors for healthy subjects in our study may be due to interindividual variability (34), resulting in a slightly higher visual sensitivity in our sample group. Lastly, the SVV misalignment of RDT of our results was always in the direction of the visual stimuli, which corresponds with previous results (47, 48).

Preliminary results showed that participants had less postural sway disturbances in presence of a tilted frame compared to the black screen condition. This adds to the literature that any reference frame, even if it deviates from the earth gravitational as in this study, provides the participant with a visual aid to maintain a more stable upright position. The perception of verticality was more disturbed in the RFT than in the SVV test, but less so than in the RDT. The literature confirms that rotating dots are a stronger visually disturbing stimulus than a tilted frame (38).

Lastly, this study aimed to identify PPPD in chronic dizziness patients by means of commonly used clinical tests. Balance and visual dependence tests were chosen, since poor balance and increased visual dependence have been reported in previous studies as indicators of PPPD (4, 6). The results show that VVAS had the most, yet limited, discriminative value. These results complement the literature which already indicates duration of momentary worsening of dizziness, head roll-tilt SVV, and the Niigata PPPD questionnaire as useful tools in identifying PPPD. More specifically, duration of momentary worsening of dizziness can distinguish between PPPD and psychogenic chronic dizziness (49), the head roll-tilt SVV can help diagnosing PPPD in chronic vestibular disorders (50), and the Niigata PPPD questionnaire is useful in patients with other vestibular disorders (not specified as chronic) to detect PPPD (5).

### Analysis of Study Strengths and Weaknesses

Strengths of this study include (1) the meticulous elimination of visual fixation points during the visual dependence tests (e.g., completely darkened room, edges of the television screen covered by a ring), (2) preliminary analysis of the RFT, RDT and postural sway while facing a tilted frame vs. rotating dots, and (3) the randomization of the visual dependence tests to eliminate a habituation effect on the results. Limitations of the study are (1) the limited sample size, (2) not having performed vestibular tests to confirm the absence of vestibular deficits in healthy persons, (3) the slightly younger age of the healthy persons, (4) the use of “best of three attempts” for the Timed Up and Go and Tandem Gait tests which may have induced a ceiling effect, and (4) the administration time of the test protocol (2.5 h) which may have caused fatigue in some patients.

### Implications for Clinical Practice and Future Research

The VVAS was the most useful clinical test for the detection of PPPD of the balance and visual dependence tests examined in this study. Both PPPD and dizzy non-PPPD patients had significantly impaired balance and increased visual dependence compared to healthy persons. This shows the importance of evaluating balance and visual dependence in patients with chronic dizziness. The clinical tests can be useful to chart the patient's individual complaint profile. This profile can then be used to accentuate certain therapy elements, for example the degree of visual desensitization training.

Further research can investigate the discriminative value of other clinical tests not discussed in this article, preferably in combination with the VVAS results.

## Conclusion

PPPD patients were more visually dependent, but did not have worse postural balance compared to dizzy non-PPPD patients. The VVAS had the most, but limited, discriminative value for identifying PPPD in chronic dizziness. In clinical practice, evaluation of balance control and visual dependence is indicated in patients with chronic dizziness, and corresponding exercises should be integrated into their exercise program in a patient-tailored way.

## Data Availability Statement

The datasets presented in this study can be found in online repositories. The name of the repository and accession number can be found at: Zenodo, https://zenodo.org/, doi: 10.5281/zenodo.6077074.

## Ethics Statement

The studies involving human participants were reviewed and approved by Medical Ethics Committees of the Antwerp University Hospital (reference number 18/586). The patients/participants provided their written informed consent to participate in this study.

## Author Contributions

All authors contributed to the outline of the research goals, the analysis/interpretation of data or editing of the manuscript, and the final reviewing and approval of the manuscript. All authors contributed to the article and approved the submitted version.

## Funding

This work was supported by the University of Antwerp (Faculty of Medicine and Health Sciences), Antwerp, Belgium.

## Conflict of Interest

The authors declare that the research was conducted in the absence of any commercial or financial relationships that could be construed as a potential conflict of interest.

## Publisher's Note

All claims expressed in this article are solely those of the authors and do not necessarily represent those of their affiliated organizations, or those of the publisher, the editors and the reviewers. Any product that may be evaluated in this article, or claim that may be made by its manufacturer, is not guaranteed or endorsed by the publisher.
