# Supplementary material for: Comparison of Clinical Balance and Visual Dependence Tests in Patients With Chronic Dizziness With and Without Persistent Postural-Perceptual Dizziness: A Cross-Sectional Study
Source: Front Neurol. 2022 May 24;13:880714. doi: 10.3389/fneur.2022.880714 (PMC9170888; doi:10.3389/fneur.2022.880714)
Supplement: Supplementary file 1 [file Data_Sheet_1.PDF]

Supplementary Material

1 Supplementary Tables

Table 1. Test conditions of the Subjective Visual Vertical test, Rod-and-Disc test, and postural sway while facing rotating dots [a]

Subjective Visual Vertical test

Test condition	*Background*	*Line*	*Screen display*
Test condition 1 (1 trial)	Black	Left tilt (-20°)	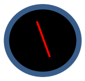
Test condition 2 (1 trial)	Black	Right tilt (+20°)	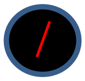
Test condition 3 (1 trial)	Black	Left tilt (-20°)	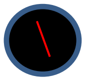
Test condition 4 (1 trial)	Black	Right tilt (+20°)	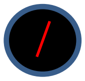

Rod-and-Disc test

Test condition	*Background*	*Line*	*Screen display*
Test condition 1 (2 trials)	CW rotating (+30°/sec)	Left tilt (-20°)	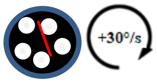
Test condition 2 (2 trials)	CW rotating (+30°/sec)	Right tilt (+20°)	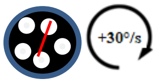
Test condition 3 (2 trials)	CCW rotating (-30°/sec)	Left tilt (-20°)	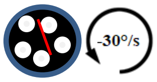
Test condition 4 (2 trials)	CCW rotating (-30°/sec)	Right tilt (+20°)	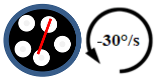

Postural sway while facing rotating dots

Test condition	*Background*	*Line*	*Screen display*
Test condition 1 (1 trial)	Black	No line displayed	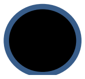
Test condition 2 (1 trial)	CW rotating (+30°/sec)	No line displayed	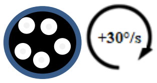
Test condition 3 (1 trial)	CCW rotating (-30°/sec)	No line displayed	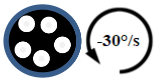

[a] Abbreviations: CCW = counter-clockwise; CW = clockwise

Table 2. Test conditions of the Rod-and-Frame test, and postural sway while facing a tilted frame

Rod-and-Frame test

Test condition	*Background*	*Line*	*Screen display*
Test condition 1 (2 trials)	Right tilted frame (+20°)	Left tilt (-20°)	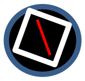
Test condition 2 (2 trials)	Right tilted frame (+20°)	Right tilt (+20°)	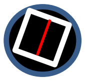
Test condition 3 (2 trials)	Left tilted frame (-20°)	Left tilt (-20°)	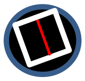
Test condition 4 (2 trials)	Left tilted frame (-20°)	Right tilt (+20°)	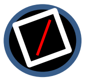

Postural sway while facing a tilted frame

Test condition	*Background*	*Line*	*Screen display*
Test condition 1 (1 trial)	Black	No line displayed	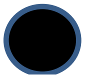
Test condition 2 (1 trial)	Right tilted frame (+20°)	No line displayed	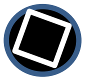
Test condition 3 (1 trial)	Left tilted frame (-20°)	No line displayed	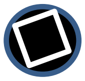

Table 3. Results on Rod-and-Frame test and postural sway while facing a tilted frame of the included participants [a, b]

Measurement tool (unit)	Mean ± SD			Statistical analyses (p-values)			
	Chronic dizziness						
	With PPPD (n = 38)	Without PPPD (n = 21)	Healthy (n = 69)	3-group comparison	With PPPD vs without PPPD	With PPPD vs healthy	Without PPPD vs healthy
RFT (°)	2.70 ± 1.77	2.99 ± 3.06	2.00 ± 1.84	0.100 †			
Postural sway while facing a tilted frame							
- COP lean (mm)	0.36 ± 8.33	0.004 ± 6.04	-0.03 ± 3.03	0.940 †			
- COP velocity (mm/s)	-0.76 ± 4.76	-1.05 ± 4.10	-1.89 ± 1.99	0.229 †			
- COP area (mm^2)	52.84 ± 301.65	-14.86 ± 161.40	-32.23 ± 64.16	0.072 †			

[a] ANOVA test (†), Independent samples t-test (‡), Chi-squared test (§), post-hoc analysis with Tukey correction (¶)
ANOVA Bonferroni cut-off: p<0.006 (**); other tests: p<0.05 (*) and p<0.001 (***)

[b] Abbreviations: COP = centre of pressure; mm = millimetres; PPPD = persistent postural-perceptual dizziness; RFT = Rod-and-Frame test; s = seconds; SD = standard deviation
